# Oxylipins Derived from PUFAs in Cardiometabolic Diseases: Mechanism of Actions and Possible Nutritional Interactions

**DOI:** 10.3390/nu16223812

**Published:** 2024-11-07

**Authors:** Duygu Ağagündüz, Özge Yeşildemir, Emine Koçyiğit, Tevfik Koçak, Buket Özen Ünaldı, Gamze Ayakdaş, Ferenc Budán

**Affiliations:** 1 Department of Nutrition and Dietetics, Faculty of Health Sciences, Gazi University, 06490 Ankara, Türkiye; 2 Department of Nutrition and Dietetics, Bursa Uludag University, Görükle Campus, 16059 Bursa, Türkiye; ozgeyesildemir@uludag.edu.tr; 3 Department of Nutrition and Dietetics, Ordu University, Cumhuriyet Yerleşkesi, 52200 Ordu, Türkiye; kocyigitem@gmail.com; 4 Department of Nutrition and Dietetics, Gümüşhane University, Gümüşhanevî Kampüsü, 29100 Gümüşhane, Türkiye; tkocak@gumushane.edu.tr; 5 Department of Nutrition and Dietetics, Faculty of Health Sciences, Afyonkarahisar Health Sciences University, 03030 Afyonkarahisar, Türkiye; buket.ozen@afsu.edu.tr; 6 Department of Nutrition and Dietetics, Acıbadem University, Kerem Aydınlar Campus, 34752 İstanbul, Türkiye; gamze.ayakdas@acibadem.edu.tr; 7 Institute of Physiology, Medical School, University of Pécs, H-7624 Pécs, Hungary

**Keywords:** oxylipins, PUFA, lipidomics, cardiometabolic diseases

## Abstract

Oxylipins are oxidized fatty acids, both saturated and unsaturated, formed through pathways that involve singlet oxygen or dioxygen-mediated oxygenation reactions and are primarily produced by enzyme families such as cyclooxygenases, lipoxygenases, and cytochrome P450. These lipid-based complex bioactive molecules are pivotal signal mediators, acting in a hormone-like manner in the pathophysiology of numerous diseases, especially cardiometabolic diseases via modulating plenty of mechanisms. It has been reported that omega-6 and omega-3 oxylipins are important novel biomarkers of cardiometabolic diseases. Moreover, collected literature has noted that diet and dietary components, especially fatty acids, can modulate these oxygenated lipid products since they are mainly derived from dietary omega-3 and omega-6 polyunsaturated fatty acids (PUFAs) or linoleic acid and α-linolenic by elongation and desaturation pathways. This comprehensive review aims to examine their correlations to cardiometabolic diseases and how diets modulate oxylipins. Also, some aspects of developing new biomarkers and therapeutical utilization are detailed in this review.

## 1. Introduction

Dietary fats have been studied as potential risk factors for cardiometabolic diseases such as obesity, diabetes, cardiovascular diseases, and nutrition-related diseases [1]. Dietary fats have impacts on the etiology of cardiometabolic diseases, on underlying pathophysiological processes [2,3]. Also, their composition and quantity, their interactions with other nutrients, and the way the food is processed and prepared are relevant [2,3]. Improvements in lipidomic analysis have made it possible to detect minor species of lipids as indicators of risk for cardiometabolic diseases [4,5].

Lipids were first identified as cell membrane structure components and a source of energy through metabolism. Moreover, bioactive lipid molecules are important cell signal transducers and regulators. Fatty acids have many chemical alterations that significantly enhance their functional range and biological effects [6,7]. The term “oxylipin” was first used in 1991 to describe oxygenated substances generated from fatty acids produced by at least one mono- or dioxygenase oxygenation process [7]. Oxylipins, as bioactive lipids, can be found in all tissues of an organism [8]. They are substantially synthesized during inflammation and oxidative stress, and they have a close relationship with omega-3 or omega-6 PUFAs within the diet [9].

Humans and other mammals lack the enzymatic capacity to convert omega-6 to omega-3 PUFAs. The concentrations of these oxylipin precursors indicate the intake of omega-3 or omega-6 PUFAs within the diet. Compounds derived from omega-3 fatty acids typically exhibit anti-inflammatory properties, while oxylipins originating from omega-6 fatty acids generally demonstrate pro-inflammatory characteristics. The modification of dietary fatty acid composition may influence the equilibrium of oxylipins, which has diverse biological impacts, by modifying the accessibility of their fatty acid precursors [10,11,12].

The most known oxylipins are the eicosanoids derived from arachidonic acid (AA) [13]. Several prostaglandins are produced from AA, and also from 20:3*n*-6 and 20:5*n*-3 [14]. Oxylipins also play a role in energy metabolism, angiogenesis, apoptosis, aging, pain, cellular adhesion, migration, proliferation, blood pressure, and vascular permeability [15,16]. Furthermore, they might influence adipose tissue function and insulin signaling via endocrine, paracrine, and autocrine processes [15]. This was relevant when the discovery of aspirin’s ability to inhibit COX enzymes and the production of their metabolites prompted extensive research into their significance in the context of various diseases. A wide range of biological processes associated with cardiometabolic health is regulated by oxylipins, including glucose homeostasis, insulin signaling, inflammation, blood pressure, energy metabolism, vascular tone, blood coagulation, bone healing, and endothelial permeability [17,18,19,20].

This comprehensive review evaluates the recent findings on the role of PUFA-derived oxylipins in glucose-lipid metabolism, and cardiometabolic outcomes. It examines particular publications published over the past five years (2019–2024) to emphasize the most recent advancements in many significant areas, including obesity, diabetes, cardiovascular diseases, non-alcoholic fatty liver disease (NAFLD), and chronic kidney disease (CKD), which threaten human health and are considered among the most significant challenges by the World Health Organization [21,22]. Prevention or treatment of these diseases by dietary modulations is a critical approach [22]. Furthermore, technological advancements and future directions of the modulation of pathogenic effects by oxylipins are elucidated.

## 2. Synthesis of Oxylipins and Their Pathways

Oxylipins are PUFA oxidation products formed by one or more mono- or dioxygen-dependent reactions. Three fundamental processes typically synthesize oxylipins. The processes involved in this process include the selection of fatty acid substrates, followed by enzymatic oxidation or non-enzymatic reactions catalyzed by free radicals, and ultimately the synthesis of metabolites [7,23]. Such metabolites are from linoleic acid (LA, C18:2*n*-6), dihomo-gamma-linolenic acid (DGLA, C20:3*n*-6), alpha-linolenic acid (ALA, C18:3*n*-3), gamma-linolenic acid (GLA), adrenic acid (AdA), AA (C20:4*n*-6), eicosapentaenoic acid (EPA, C20:5*n*-3), and docosahexaenoic acid (DHA, C22:6*n*-3) [24].

The precursors of oxylipins derived from PUFAs can be acquired by dietary intake or elongation and desaturation of LA and ALA into longer-chain PUFAs. Phospholipase A2 releases PUFAs from phospholipids in the membrane, which are further oxygenated by more than 50 enzymes. These enzymes are classified as cytochrome P450s (CYPs), lipoxygenases (LOXs), cyclooxygenases (COXs), and enzymes involved in free radical-induced photodegradation, autooxidation, and peroxidation [25,26].

Oxylipin synthesis starts with cell activation. The synthesis of oxylipins occurs through a sequence of reactions facilitated by enzymes belonging to the phospholipase A2 superfamily. This process begins with the removal of fatty acids from phospholipids in the cell membrane, leading to the liberation of precursor PUFAs at the sn-2 position of the membrane phospholipids by cytosolic phospholipase A2 (cPLA2) [27]. The three main enzymatic pathways involved in oxylipin synthesis are catalyzed by COX, LOX, and CYP oxidase enzymes. In general, the process of PUFA oxygenation is started by COXs, LOXs, and, to a lesser degree, CYPs [7].

**Figure 1:**
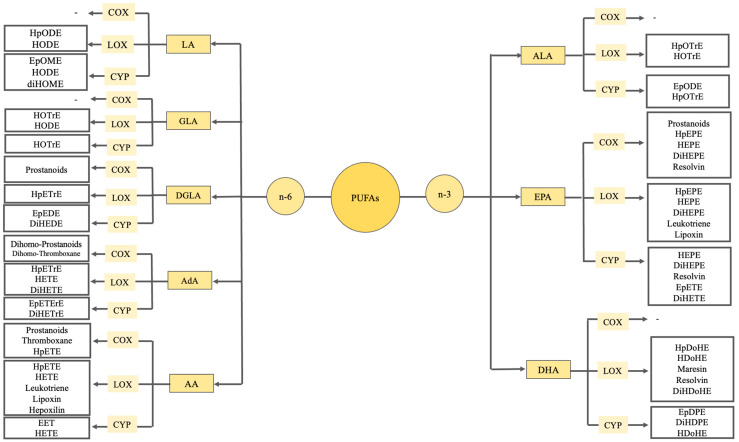
Summary of oxylipins synthesized from PUFAs. PUFA: Polyunsaturated fatty acids, LA: Linoleic acid, GLA: γ-linolenic acid, DGLA: Dihomo-γ-linolenic acid, AdA: Adrenic acid, AA: Arachidonic acid, ALA: α-Linolenic acid, EPA: Eicosapentaenoic acid, DHA: Docosapentaenoic acid, COX: Cyclooxygenase, LOX: Lipoxygenase, CYP: Cytochrome P450, HpODE: hydroperoxy-octadecadienoic acid, HODE: Hydroxy-octadecadienoic acid, EpOME: Epoxy-octadecenoic acid, diHOME: Dihydroxy-octadecenoic acid, HOTrE: Hydroxy-octadecatrienoic acid, HpETrE: Hydroperoxy-eicosatrienoic acid, EpEDE: Epoxy-eicosadienoic acid, DiHEDE: Dihydroxy-eicosadienoic acid, HETE: Hydroxy-eicosatetraenoic acid, DiHETE: Dihydroxy-eicosatetraenoic acid, DiHETrE: Dihydroxy-eicosatrienoic acid, HpETE: Hydroperoxy-eicosatetraenoic acid, EET: Epoxyeicosatrienoic acids, HpOTre: hydroperoxy-octadecatrienoic acid, EpODE: Epoxy-octadecadienoic acid, HpOTrE: Hydroperoxy-octadecatrienoic acid, HpEPE: Hydroperoxy-eicosapentaenoic acid, HEPE: hydroxy-eicosapentaenoic acid, DiHEPE: dihydroxy-eicosapentaenoic acid, EpETE: Epoxy-eicosatetraenoic acid, HpDoHE: Hydroperoxy-docosahexaenoic acid, HDoHE: Hydroxy-docosahexaenoic acid, DiHDoHE: Dihydroxy-docosahexaenoic acidEpDPE: Epoxy-docosapentaenoic acid, DiHDPE: Dihydroxy-docosapentaenoic acid.

Oxylipins derived from C18 PUFAs such as LA and ALA are octadecanoids; eicosanoids from C20 fatty acids such as ARA, DGLA, and EPA; classic eicosanoids such as PGs, thromboxans, and leukotriens; and docosanoids of C22 fatty acid such as AdA, DPA, and DHA [6]. Oxylipins include a variety of oxygenated derivatives of DHA and EPA, with certain enantiomers referred to as “special pro-solution mediators” (SPMs) because of their putative function in reducing inflammation. Resolvins (Rvs), maresins (MaRs), and protectins (PDs) are SPMs derived from DHA [28]. Figure 1 shows a summary of oxylipins synthesized from PUFAs.

### 2.1. Cyclooxygenase

The first pathway of oxylipin synthesis involves COX enzymes. These enzymes convert PUFAs to prostanoids. COXs are enzymes that contain heme and can perform both oxygenase and peroxidase functions. They catalyze the initial oxygenation of non-esterified fatty acids (NEFA) to produce prostaglandin H (PGH), and this intermediate is then metabolized to prostanoids such as prostaglandin D, E, and F series (PGD, PGE, and PGF), prostacyclins (PGI), and thromboxanes (TXs) [29]. When prostanoids are synthesized and released, they attach to intracellular effectors like peroxisome proliferator-activated receptors (PPAR) or G protein-coupled receptors (GPCRs) on the cell surface to cause an effect [30]. Prostanoids are regarded as “local hormones”. They are mostly synthesized in the tissues, where they also have an impact [31].

There are two main COX isoforms in humans: COX-1 and COX-2. COX-1 is a constitutive enzyme whereas COX-2 is an inducible form [7]. Nearly all tissues consistently produce COX-1, with blood vessels, platelets, mesothelial cells, smooth muscle cells, and interstitial cells producing it at higher concentrations [32]. Its levels remain constant throughout the cell cycle [33]. It plays a role in the regulation of synthesizing PGs, which control physiological processes [24]. Normally, just a few tissues—the blood vessels, brain, kidney, lung, and thymus—express COX-2 to enhance local blood circulation. When inflammation is triggered by substances such as endotoxins, cytokines, tumor promoters, and certain lipids, COX-2 is first expressed at low levels and can then be quickly produced [34].

COX isoforms exhibit differences in substrate selectivity, expression, and tissue distribution [24]. In general, COX-2 has a wider range of PUFAs than COX-1. However, AA is the most preferred substrate for both isoforms [35]. This catalytic process converts AA to PGH2, a precursor to additional PGs and TXs. The catalytic process also accepts other PUFAs such as DGLA and EPA as substrates [36].

### 2.2. Lipoxygenase

LOXs are involved in the second pathway of oxylipin synthesis. They catalyze the formation of hydroperoxides, which are further reduced by glutathione peroxidases [29]. LOXs are multifunctional enzymes that can catalyze three distinct types of reactions: the synthesis of epoxy leukotrienes through the leukotriene synthase process; the hydroperoxidation of lipids to keto lipids; and the dioxygenation of lipids to hydroperoxides [24].

The position of the hydroxy and hydroxy fatty acids that each LOX enzyme forms from AA is the traditional method of classifying these enzymes [37]. The human genome has six functional LOX genes that are expressed in different tissues. The following are the specific types of LOX enzymes: 5-LOX, 12-LOX, 15-LOX type 1, 15-LOX type 2, 12 R-LOX, and epidermal LOX [38]. The association of all LOXs found in the human genome with membranes is calcium dependent. The regulation of each enzyme is not clear. Nevertheless, 5-LOXs require the aid of protein partners to facilitate movement across the membrane and carry out their functions. On the other hand, 15-LOXs only require a partner protein to effectively catalyze the oxygenation of PL-esterified PUFA [39].

5-LOX is the key enzyme in the formation of leukotrienes [40]. Leukotriene synthesis is initiated by a coordinated two-step process. The initial step involves the dioxygenation of C5 into 5S-hydroperoxy-6t,8c,11c,14c-eicosatetraenoic acid (5-HPETE), typically occurring in the AA state [24]. The following step requires the presence of two helper proteins. The proteins mentioned are coactosin-like protein (CLP) and 5-lipoxygenase activating protein (FLAP). Through these two proteins, 5-LOX catalyzes the conversion of arachidonic acid into 5-HPETE. Afterwards, 5-HPETE can either be reduced by glutathione peroxidase to form 5-HETE or converted by 5-LOX into leukotriene A4 (LTA4), which is a precursor for other leukotrienes [28].

Each LOX isoform has distinct substrate preferences. 5-LOX substrates consist of epoxy alcohols that are obtained from AA, EPA, and DHA. AA is crucial in the process of producing leukotrienes, while EPA and DHA serve as the precursors for the synthesis of E and D series Rvs [41]. EPA is also oxygenated into leukotrienes. The substrates of 12S-LOX include AA, DGLA, EPA, and DHA. 15-LOXs mostly use EPA and DHA as substrates [39].

### 2.3. Cytochrome P450

The third oxylipin synthesis pathway involves CYP mixed-function oxidase enzyme activity as monooxygenases. It catalyzes epoxidation, hydroxylation, and allylic oxidation, which convert PUFAs into lipid mediators that possess a wide array of cellular and systemic biological functions [42]. As oxylipins are synthesized through other processes, they exert their effects via interacting with specific receptors such as PPAR, aryl hydrocarbon receptor (AhR), and GPCRs, or by binding to other oxylipin receptors. Additionally, they have the ability to infiltrate cells and regulate the activity of transcription factors and ion channels [10].

CYPs are a group of monooxygenases that include heme and are found in all domains of life. There are 57 functional CYP genes in humans, and these genes produce proteins that may be classified into 18 families and 41 subfamilies based on their amino acid sequence [43].

The CYP enzymes responsible for the synthesis of oxylipins may exhibit either epoxygenase or ω-hydroxylase activity [44]. EPA is the substrate preferred for most CYP isoforms. Epoxyeicosatetraenoic acid (EpETE) is produced by epoxygenase, while hydroxyeicosapentaenoic acid (HEPE) is produced from EPA via ω-hydroxylase activity [42]. DHA and AA are metabolized at similar rates. Epoxyeicosatrienoic acid (EET) from AA is produced by epoxygenase and HETE are produced by ω-hydroxylase activity. From DHA and epoxydocosapentaenoic acid (EDP) is produced by epoxygenase and HDoHE is produced by ω-hydroxylase activity [7].

## 3. The Role of Oxylipins in Cardiometabolic Diseases and Their Specific Dietary Modulations

AA, EPA, and DHA are lipid mediators that exert significant effects on the inflammatory response [15]. Namely, oxylipins derived from EPA or DHA are specialized pro-resolving mediators that exert anti-inflammatory effects by influencing signal transduction [15]. Oxylipins can act as inflammatory or anti-inflammatory molecules through their connection to both PPARs and GPCRs [45]. Omega-6 oxylipins are known as pro-inflammatory, whereas omega-3 oxylipins are anti-inflammatory [46]. However, some omega-6 oxylipins may show anti-inflammatory effects [47]. Though omega-6 oxylipins have vasoconstrictor, proliferative, and pro-thrombotic properties, which are implicated in developing cardiometabolic diseases, omega-3 oxylipins have the opposite effects [48]. Specifically, AA-derived mono-hydroxides, namely HETEs are associated with obesity, cancer, thrombogenesis, cardiovascular diseases, and diabetes. Conversely, EPA-derived oxylipins, primarily HEPEs, and series-3 PGs, provide anti-inflammatory and cardioprotective functions [49].

Chronic low-grade inflammation, characterized by phenotypic alterations in immune cell subsets and histological alterations in tissues, is a crucial component of cardiometabolic disorders. The afflicted tissues eventually release pro-inflammatory cytokines, acute phase proteins, pro-inflammatory lipids, and other inflammatory mediators into the bloodstream, turning tissue-based low-grade inflammation into a systemic inflammatory disease [50]. Attenuating the systemic inflammatory response may prevent or reduce the severity of cardiometabolic diseases [22]. Therefore, it is important to identify local inflammatory biomarkers before low-grade inflammation leads to systemic diseases. Several blood-based biomarkers, such as acute phase proteins, cytokines, and chemokines, are effectively used, which may reflect distinct stages in chronic low-grade inflammation [50].

**Figure 2:**
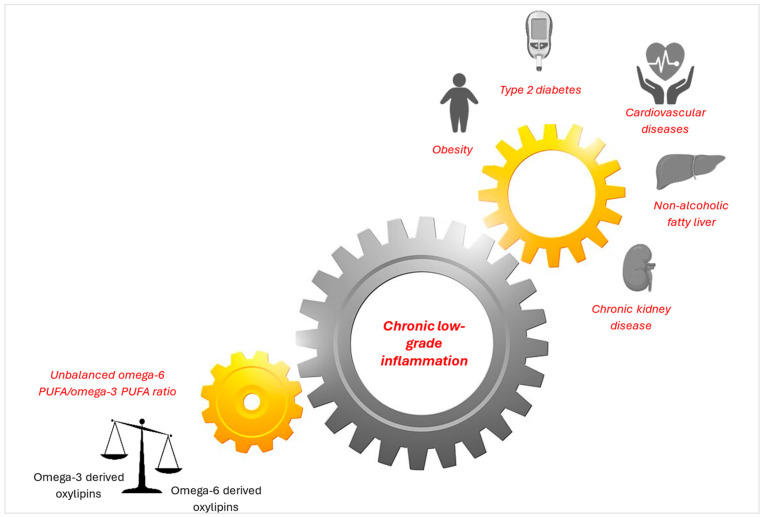
The role of chronic low-grade inflammation in revealing the relationships between oxylipins and cardiometabolic diseases.

However, there are also more innovative biomarkers like oxylipins, which are oxidation products of PUFAs. Recently, omega-3 and omega-6 oxylipins have attracted researchers’ attention as inflammatory biomarkers, which are some of the factors that lead to the development of various cardiometabolic diseases [49]. Figure 2 shows the role of chronic low-grade inflammation in revealing the relationship between oxylipins and cardiometabolic diseases.

The profile of lipid metabolites and mediators derived from omega-3 and omega-6 PUFAs is expected to change due to the disruption of PUFA metabolism in cardiometabolic diseases [21]. Altered oxylipin profiles have been described in many inflammation-related diseases, including obesity [51], type 2 diabetes [20], metabolic syndrome [52], NAFLD [53], cardiovascular diseases [54], and CKD [55]. A balanced omega-6/omega-3 oxylipin ratio may have protective effects against obesity, insulin resistance, metabolic syndrome, hepatic steatosis, and potentially cancer, according to preclinical research [56,57]. Similarly, human studies have suggested that an imbalance between omega-3 and omega-6 fatty acids may contribute to an elevated risk of type 2 diabetes [58], hyperlipidemia [59], and liver diseases [60]. Jurado-Fasoli et al. (2023) determined that a high omega-6/omega-3 oxylipin ratio was associated with an unfavorable cardiometabolic profile (i.e., high glucose and lipid levels) [49]. Furthermore, it has been shown that plasma levels of omega-6 oxylipins are positively correlated with adiposity and a worsened cardiometabolic profile in young adults without chronic diseases despite the opposite effects of omega-3 oxylipins. It has been noted that omega-6 and omega-3 oxylipins are better predictors of the early development of cardiometabolic risk factors than traditional inflammatory markers [i.e., interferon-gamma (IFN-γ) and tumor necrosis factor-alpha (TNF-α)] [52]. Therefore, oxylipins may be one of the critical markers for identifying people with an increased cardiometabolic risk and early treatment of them [49].

**Figure 3:**
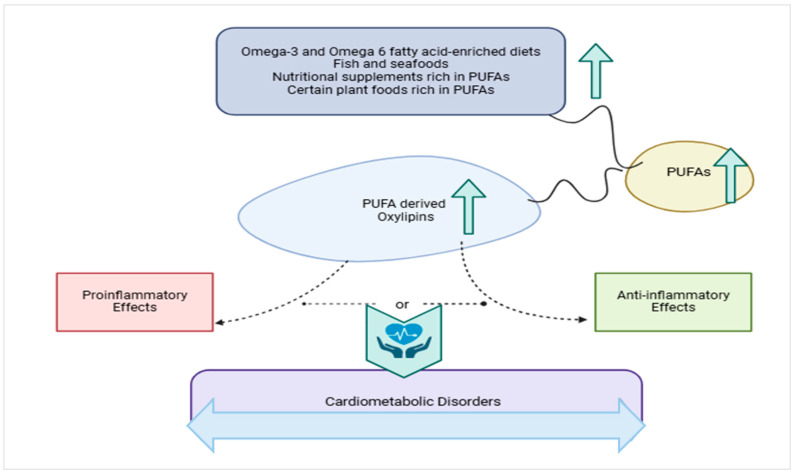
Possible dietary modulation of PUFA-derived oxylipins and their cardiometabolic effects which depends on consumed PUFA types and amounts. PUFAs: Polyunsaturated fatty acids.

Dietary fatty acids significantly affect human tissues’ oxylipin profiles and their cardiometabolic effects. Dietary fatty acids can modulate PUFA-derived oxylipin profiles and their cardiometabolic effects regarding oxylipins in the body depending on consumed PUFA types and amounts (Figure 3). PUFAs are consumed through the diet via some foods and nutritional supplements, and their profile in human tissues is associated with consumed quantity. For example, the ratio between dietary ALA:LA and renal ALA:LA concentrations was 0.96 [8]. In an animal study, rats were fed with control, high LA, or high LA and high ALA (LA + ALA) diets for 6 weeks. Dietary LA increased omega-6 oxylipins’ concentration in the bloodstream. The addition of ALA to the diet generally returned omega-6 oxylipins near to the control levels and increased some omega-3 oxylipins [48]. Some studies reported that the concentration of circulating oxylipins increased about 2–6 h after consumption of omega-3 fatty acid-enriched milkshakes or supplements [61,62]. Also, long-term fish oil supplementation studies observed changes in oxylipin levels in the circulation [63,64,65]. Additionally, it can be observed that oxylipin profiles change after introducing diets that are not explicitly aimed at modulating PUFA levels, such as low-calorie or low-fat diets [66]. This reveals the importance of dietary modulations in preventing and treating cardiometabolic diseases by changing the endogenous oxylipin profile [49].

As shown on the left in Figure 2, an unbalanced increase in dietary omega-6 polyunsaturated fatty acids (PUFA) and a decrease in omega-3 PUFA are among the most common triggers of chronic low-grade inflammation via the oxylipins pathway. As shown on the right, the consequences of chronic low-grade inflammation include obesity, type 2 diabetes, cardiovascular diseases, non-alcoholic fatty liver disease, and chronic kidney disease.

### 3.1. Obesity

Obesity is defined as abnormal or excessive fat accumulation that poses a health risk. The burden of obesity incidence is rapidly growing worldwide [67]. The World Obesity Federation predicts that one billion people globally will be living with obesity by 2030, including one in five women and one in seven men [68].

Obesity is related to chronic low-grade inflammation within adipose tissue [69]. As the adiposity of tissues increases, various metabolic tissues become inflamed, including white adipose tissue (WAT), liver, skeletal muscle, brain, and pancreas. WAT expansion increases immune cells’ quantity and also activates them, including pro-inflammatory CD4+ and CD8+ T-cell subsets, CD19+ B cells, and M1-like macrophages. Additionally, it causes pro-inflammatory cytokine secretion and oxidative stress. Ultimately, these modifications result in an inflammatory microenvironment [70], increasing the risk of metabolic diseases such as type 2 diabetes, hypertension, NAFLD, kidney diseases, cardiovascular diseases, and obesity-related malignancies [71].

At a molecular level, inflammation in obesity is controlled by the metabolism of PUFAs and their oxidized lipids, oxylipins [67]. Oxylipins are involved in the initiation and the cessation of inflammatory processes [72]. Although there are notable exceptions, omega-6 oxylipins have pro-inflammatory functions that play a role in the onset and progression of obesity, while omega-3 oxylipins show anti-inflammatory effects [52]. They may also regulate several metabolic processes associated with adipose tissue, including adipogenesis and energy utilization [72,73]. Oxylipins from omega-6 PUFAs have pro-inflammatory properties that enhance immune cell infiltration and activation, modify lipid metabolism, and stimulate WAT expansion. Omega-3 PUFAs, which oxidize to form SPMs), regulate leukocyte infiltration and reduce pro-inflammatory cytokine expression [74]. Thus, omega-3 PUFAs are expected to exert an anti-obesity effect [30].

It is a known fact that the oxylipin profile changes pathologically in obesity [71]. Additionally, compared to individuals who are overweight or obese, people with severe obesity have significantly greater plasma levels of total oxylipin and fatty acids [51]. There is compelling evidence from studies with animals and humans that obesity leads to an imbalanced signature of oxylipins [52,66,74,75,76,77,78,79,80,81,82,83,84,85]. Indeed, in a study with type 2 diabetes mellitus (T2DM)-afflicted obese women compared to obese women of the same age and BMI but without T2DM in the subcutaneous white adipose tissue (sWAT), the pro-inflammatory 5-LOX, DHA, AA, and the gene expression of AA positively correlated macrophage marker CD68 level was increased [86]. Ample evidence has suggested that among young people obesity increased plasma levels of omega-6 oxylipins and lowered plasma levels of omega-3 oxylipins [52,86]. In a study, the omega-6 oxylipins such as AA, LA, and DGLA has been detected at an increased level in obesity, leading to the formation of pro-inflammatory lipid mediators [87].

Furthermore, it was reported that obese patients had higher levels of hydroxy-AA metabolites (5-, 11-, 12-, 15- and 20-HETE), which are often associated with inflammatory conditions, than those of normal control subjects [52,66,78]. Pickens et al. (2017) showed that increased 5-, 11-, and 15-HETE concentrations were positively associated with body mass index (BMI), waist circumference, and serum leptin in Caucasian men [78]. Increases in the pro-inflammatory 5-HETE may play a role in the pathological alterations of obese adipose tissue, which may influence PUFA peroxidation by promoting oxidative stress and raising reactive oxygen species [78]. Therefore, it is thought that 5-HETE may be a marker of a low-grade inflammatory state in obesity [66]. 11-HETE and 15-HETE generally contribute to the development of diabetes, cardiovascular diseases, and some cancers [78,88]. 15-LOX inhibitors are even already used to combat some of these diseases [88]. Although evidence linking 11-HETE and 15-HETE with obesity is limited, it is not surprising that these oxylipins are a biomarker of obesity-related pathology since obesity is also closely associated with cardiovascular diseases and cancer [78]. 12-HETE, a 12-LOX product, is well-documented to induce inflammation and leukocyte infiltration in obese adipose tissue [89]. An animal study determined that 20-HETE, a lipid mediator whose levels significantly increased in animals fed fat-rich diets, contributed to rapid weight gain [90].

Moreover, PGE2 is the predominant PG produced by adipose tissue and plays a vital role in the pathogenesis of various inflammatory diseases [91]. Regarding obesity, PGE2 has been shown to play a role in regulating adipose tissue functions and developing obesity. In this context, it was found that PGE2 and COX-2 levels increased in the adipose tissue of obese patients [75,76]. It has also been suggested that PGE2 exhibits anti-lipolytic effects that lead to increased adipose tissue mass [91]. Likewise, it was determined that PG signaling was impaired, and PGD2 levels were permanently increased in mice fed a high-fat diet [92]. PGI2 also increases adipogenesis via the PGI2 receptor. PGI synthase knockout mice fed a high-fat diet showed decreased body weight gain compared to the wild-type mice [77]. 15-deoxy-Δ12,14-prostaglandin J2 (15d-PGJ2), a metabolite derived from PGD2, has a pro-adipogenic effect [93].

A report showed that 5-LOX expression upregulated in the adipose tissue of obese patients [86]. In addition, the activity of 5-LOX is essential in chronic and acute inflammation [94]. Inhibition of 5-LOX has reduced hepatic macrophage infiltration in obese mice fed a high-fat diet [95].

Leukotriene B4 (LTB4), a lipid mediator derived from AA by the sequential action of 5-LOX, is elevated in obese mice. It is also a potent leukocyte chemoattractant and activator promoting chronic inflammation in WAT [79,80]. According to a study, blocking the LTB4 receptor in obese mice altered the inflammatory profile and decreased peritoneal macrophage chemotaxis to target organs [79]. In another study, it was observed that excessive production of LTB4 triggered inflammation by causing increased chemotaxis of circulating B cells to WAT [96]. Similar to animal studies, it was found that leukocytes from obese people exhibited a four-fold increase in LTB4 [84].

EETs are hydrolyzed by soluble epoxide hydrolase (sEH) to dihydroxy-eicosatrienoic acids (DHETs), which are only biologically active [97]. It has been reported that EETs may mediate anti-inflammatory mechanisms by blocking the activation of nuclear factor kappa B (NF-κB) and reducing TNF-α. It has been demonstrated that stimulating adipogenesis in differentiated 3T3-L1 cells (in vitro) and adipose enlargement in mice given a high-fat diet (in vivo) dysregulates the CYP epoxygenase pathway, which significantly lowers the amounts of EET generated from adipose tissue [98]. However, Jurado-Fasoli et al. (2022) noted that plasma levels of 14,15-epoxy-eicosatrienoic acid (14,15-EpETrE) were positively correlated with adiposity [52]. This result is also supported by the fact that 14,15-EpETrE levels are lower in fat-1 transgenic mice, which are protected against obesity when exposed to a high-fat diet [57].

AA-derived 15-F2t-isoprostane (15-F2t-IsoP), a classic biomarker of oxidative stress, has been linked to a 23-fold increase in individuals at risk of cardiometabolic disease compared with healthy individuals [93]. Additional cross-sectional research have exhibited that F2t-IsoP metabolites, namely 2,3-dinor-5,6-dihydro-15-F2t-IsoP, are a more sensitive marker than 15-F2t-IsoP in assessing obesity [99,100].

According to data from the Shanghai Women’s Health Study, a high concentration of urinary 2,3-dinor-5,6-dihydro-15-F2t-IsoP was positively correlated with an elevated BMI [101]. Bioactive oxidized LA metabolites in the LOX pathway, 9-HODE, and 13-HODE, have often been related to oxidative stress, inflammation, and numerous pathological and physiological conditions [102]. However, data on the levels of 9-HODE and 13-HODE in obesity are complicated and inconsistent. Liakh et al. (2022) found no difference in 13-HODE serum levels between obese and lean people [94]. Some authors have reported very high levels of 13-HODE in low-density lipoprotein (LDL) in severely obese compared with healthy subjects [103]. In contrast, others have emphasized lower serum 13-HODE levels in obese than in lean individuals [78]. Hernandez-Carretero et al. (2018) determined that 9-HODE and 13-HODE levels increased in obese compared to lean mice [92].

It is suggested that 12,13-dihydroxy-9Z-octadecenoic acid (12,13-diHOME), another LA-derived oxylipin, may regulate energy metabolism as a thermogenic metabolite [104]. A decreased visceral and total fat mass was linked to a plasma 12,13-diHOME concentration in a population of 2248 people [19]. Likewise, Lynes et al. (2017) found a substantial inverse correlation between BMI and plasma levels of 12,13-diHOME [81].

LA is converted to GLA by the Δ6 desaturase enzyme, and GLA is elongated to form DGLA, the precursor of AA. Elevated Δ6 desaturase activity in obesity may increase DGLA levels [105]. A study conducted on diabetic patients showed that serum DGLA levels were positively correlated with waist circumference, body fat percentage, and BMI [106]. Another study also reported that DGLA levels were significantly higher in overweight or obese subjects than in controls [107]. Additionally, a human study found that the concentration of 15-hydroxy-eicosatrienoic acid (15-HETrE) from DGLA was positively associated with BMI [66]. Although it is known that 15-HETrE inhibits cancer cell growth, prevents lung fibrosis, and has anti-inflammatory activity on the skin, its effect on obesity is not fully understood [10]. However, many studies have determined that 15-HETrE levels increase in obese individuals [52,66]. It has been reported that omega-3 PUFA-derived EPA, DHA, and ALA oxylipins were reduced in obese mice and people [74,82,83,84,85].

5-HEPE, an EPA metabolite, is critical in improving body energy metabolism and browning of WAT. Zong et al. (2023) fed mice with a high-fat diet for 12 weeks and then injected them intraperitoneally every other day with 5-HEPE for 4 additional weeks. This study revealed that 5-HEPE alleviated high-fat diet-induced obesity, which was associated with decreased subcutaneous fat and epididymal fat index and increased brown fat. Overall, 5-HEPE promotes brown adipose tissue (BAT) activation and browning of WAT by upregulating UCP-1 genes and protein expression [108]. Leiria et al. (2019) also found that the levels of 12-HEPE, synthesized from EPA through the 12-LOX pathway, were significantly lower in overweight and obese than in lean subjects. They suggested that 12-HEPE may reduce the risk of obesity by regulating energy homeostasis and fuel utilization [72].

Potent oxylipins that actively resolve inflammation, protect organs, and drive tissue regeneration—SPMs biosynthesized from EPA and DHA, such as Rvs, PDs, MaRs—are strongly linked to obesity [67,84]. SPMs and their precursors have been shown to significantly decrease in response to obesity in various mouse tissues, including WAT, liver, bone marrow, and spleen [83,109]. In murine models, obesity significantly decreased levels of 17-hydroxy-docosahexaenoic acid (17-HDHA), a precursor of RvD1 derived from DHA, in WAT [83]. In another study, levels of 14-HDHA were lower in the plasma, serum, and leukocytes of obese than in healthy individuals [84]. Leiria et al. (2019) concluded that 14-HDHA levels were lower in overweight and obese than in lean people [72]. Further, it has been shown that increasing the bioavailability of SPMs can minimize inflammation and mediate therapeutic effects [110]. Interestingly, cold exposure or β3-adrenergic stimulation increases the biosynthesis of MaR2 in BAT. This reduces circulating levels of TNF-α and increases the levels of hepatic monocytes expressing triggering receptors expressed on myeloid cells 2 (TREM2), an anti-inflammatory protein [111].

The mechanisms by which obesity reduces oxylipin levels derived from EPA and DHA have not yet been demonstrated directly. In an experiment using cultured leukocytes isolated from individuals with morbid obesity, it was shown that 15-LOX and 5-LOX activities, which are necessary for the synthesis of omega-3 oxylipins, were impaired [84]. However, further studies are required to define the pathways. The role of oxylipins in obesity is shown in Table 1.

There are few studies on the relationship between weight loss and oxylipin levels in obese subjects [51,112,113]. For example, Moeller et al. (2016) studied 42 obese adults in an 8-week weight loss treatment program. They observed that levels of many oxylipins remained unchanged before and after weight loss. However, they highlighted some AA-derived oxylipins (5-, 8-, 12-HETE) decreased after weight loss intervention [112]. In another study, plasma samples were collected from 28 obese and diabetic women before and 3 months after Roux-en-Y gastric bypass. While there was no significant change in the levels of EPA and DHA oxylipins, the levels of AA and its oxylipins, TXB2 and PGD2, increased [15]. Liakh et al. (2022) also identified a significant decrease in omega-6 oxylipins and an increase in omega-3 oxylipins after weight loss following bariatric surgery [94]. Although increased pro-inflammatory oxylipin production is considered an essential feature of obesity and its related comorbidities [114], the response to weight loss varies. Plasma oxylipins may not always return to normal levels, even when plasma fatty acid levels rarely may [51].

Importantly, dietary modulations have excellent potential to alter the endogenous oxylipin profile and, through this mechanism, reduce the risk of obesity [69]. Dietary lipid intake modulation may hinder obesity and other cardiometabolic diseases by increasing anti-inflammatory oxylipins [69]. Animal and human studies have suggested that oxylipins may be an independent factor exacerbating inflammation and obesity caused by high-fat diets. Therefore, reducing dietary fat intake is an opportunity to prevent obesity through oxylipins. On the other hand, diet types, especially the Western diet, characterized by high fat content contribute to the development of obesity and obesity-mediated inflammation [45]. Excessive consumption of high-fat diets has undoubtedly exacerbated the obesity epidemic [115]. High-fat diets induce a shift from normal triglyceride synthesis to lipid mediators that can cause endoplasmic reticulum stress and lipotoxicity [116]. In addition, following a high-fat diet, inflammation develops in peripheral tissues such as the liver, adipose tissue, skeletal muscle, and intestine [115].

One of the mechanisms underlying the harmful effects of high-fat diets may be the imbalance between pro-inflammatory and anti-inflammatory oxylipins. In a double-blind crossover study, high-fat (63 g/d) and high-calorie (1300 kcal/d extra) feeding in healthy men impaired body weight, BMI, waist circumference, cytokine profile, and oxylipin levels [117]. A high-fat diet has been shown to increase the levels of AA-derived PGD2, PGE2, and PGF2 in both WAT and the liver. It has been stated that this may promote chemotaxis by elevating local cytokine release and endothelial expression of adhesion molecules to facilitate immune cell infiltration [73]. Furthermore, a high-fat diet has reduced levels of anti-inflammatory oxylipins, such as 18-HEPE, a precursor to Rvs of the E series, and 17-HDHA in the liver [82].

Hypothalamic RvD2 production has also been reduced in mice fed a high-fat diet [118]. Similarly, an animal study found that mice fed a high-fat diet showed lower levels of 12-HEPE and 18-HEPE than the control group [109]. The fundamental mechanism, according to some claims, is that 18-HEPE can prevent macrophages from responding in a pro-inflammatory manner [119]. Additionally, 18-HEPE may exert its effects through Rvs of the E series, which regulate inflammatory responses by inhibiting LTB4 signaling through neutrophils, monocytes, and macrophages [67].

Moreover, the protective effects of dietary omega-3 fatty acids on obesity are generally attributed to their lipid-lowering and anti-inflammatory effects [47]. Changes in adipokine release, altered gene expression in adipose tissue, adipokine-mediated or adipokine-related pathways, modifications in carbohydrate metabolism, appetite suppression, increased fat oxidation, increased energy expenditure, and epigenetic actions are the driving mechanisms behind these effects [120].

The benefits of consuming omega-3 PUFAs for obesity are well known. Still, it is unclear whether these fatty acids or their oxygenated metabolites, oxylipins, are responsible for their beneficial effects [71]. As mentioned above, omega-3 fatty acids improve metabolic health by reducing obesity-associated inflammation. It is stated that it achieves these effects partly through conversion to oxylipins. Animal studies have shown that dietary supplementation with oils rich in omega-3 fatty acids reduces inflammation and the progression of obesity [47,73,84,121,122]. Onodera et al. (2017) found that EPA intervention increased the number of T regulatory cells in the epididymal adipose tissue of db/db mice [121]. In an animal study, intracerebroventricular treatment with DHA-derived RvD2 reduced visceral adipose tissue and reversed hypothalamic leptin resistance [118]. A study conducted in obese mice found that krill oil supplementation for 28 weeks increased concentrations of EPA and DHA-related oxylipins in WAT and liver, including 18-HEPE, RvE2, 14-HDHA, and 17-HDHA. Simultaneously, krill oil supplementation decreased levels of AA-derived oxylipins, like HETEs, PGD2, PGE2, PGF2α, and TXB2. This study has revealed that fatty acid and oxylipin composition of adipose tissue and liver can be strongly modulated by krill oil during the development of obesity [73]. It has been reported that 17-HDHA treatment reduces WAT inflammation, increases adiponectin expression, and improves glucose tolerance in obese mice [82]. Heat-producing brown or beige adipocytes are thought to be effective against cardiometabolic diseases, including obesity.

The management of BAT has excellent therapeutic potential for preventing and treating obesity and related diseases [21]. Recently, researchers have discussed the role of dietary omega-3 PUFAs in the browning of WAT. They have claimed that the induction or activation of thermogenic adipocytes may be modified by the oxylipins produced in response to dietary intervention [123]. Physiologically, BAT thermogenesis is the outcome of adrenergic activation of lipolysis by brown adipocytes, which in turn triggers the mobilization and accumulation of NEFA in adipocytes. Dietary omega-3 fatty acid supplementation has been demonstrated in numerous rodent studies to improve BAT NEFA oxidation, thermogenesis, UCP-1 expression, and adipose tissue lipolysis [124,125,126,127,128]. In an animal study, mice were fed chow diets supplemented with omega-3 or omega-6 PUFAs in order to attain a dietary omega-6/omega-3 ratio of 30 or 3.7, respectively. Following 11 weeks of feeding, the expression of UCP-1 was more pronounced in BAT and WAT of omega-3-supplemented mice. This impact is thought to be caused by PGF2α generated from AA, which is produced at a lower level in the tissues of mice. PGF2α is a thermogenesis-dependent UCP1 negative regulator [123].

These in vivo results are also supported by in vitro findings. Treatment with EPA has increased the expression of thermogenic genes in mice brown and white pre-adipocyte cells [129]. Similarly, EPA intervention has elevated UCP-1 expression in human adipose tissue-derived stem cells, whereas the same effect has not been observed in the DHA treatment [130]. Dietary intake of omega-3 fatty acids, especially EPA, affects UCP1-positive cells beneficially. This implies that dietary changes may have an impact on the endogenous oxylipin profile and the fatty acid content of membranes [123].

In spite of the strong anti-inflammatory effects observed in studies on animals [47,73,84,121,122], the results of omega-3-rich oil supplementation in obese humans are complicated [74,131,132,133]. In a randomized, double-blind, crossover clinical trial, obese women aged 20-51 were supplemented with 4 g/day of ALA or DHA for 4 weeks using ALA-rich flaxseed or DHA-rich fish oil. After 28 days of fish oil supplementation, plasma levels of all oxylipins produced from DHA (3.8-fold) and EPA (2.7-fold) were greater than at the baseline visit. Following the addition of flaxseed oil, oxylipins remained unchanged. No supplement changed the levels of plasma cytokines. However, by the conclusion of the fish oil intervention, adiponectin levels rose by 1.1 times [47]. In a recent study, 23 obese subjects consumed 2 g/d of a marine oil supplement enriched with 18-HEPE, 14-HDHA, and 17-HDHA for 28–30 days. After the intervention, some AA-derived HETEs decreased, while RvE1 and MaR1 concentrations increased by 3.5- and 4.7-fold, respectively [133]. Gabbs et al. (2021) reported that individuals with a healthy weight supplemented with DHA-rich oil experienced an increase in numerous oxylipins generated from DHA and EPA [134]. Likewise, many clinical studies have indicated that plasma DHA and EPA-derived oxylipin levels enhanced following fish oil supplementation [64,135]. It has been observed that EPA and DHA-derived oxylipins increased after omega-3 PUFA-enriched milkshake consumption in lean and obese subjects [61].

On the contrary, some studies have determined no substantial increase in DHA-derived oxylipin levels after administering omega-3 PUFA-enriched marine oils in obese people [74,133]. A randomized clinical trial showed that marine oil supplementation enormously increased oxylipin levels in the subcutaneous WAT of lean individuals but not in obese patients [74]. These findings suggest that directly administering oxylipins to obese people may be necessary because the enzymes that synthesize oxylipins from omega-3 PUFAs are likely ineffective [67]. Nevertheless, increasing the concentration of circulating omega-3 oxylipins and their metabolic intermediates by specific dietary or pharmacological modulations may be therapeutic in preventing or treating obesity-related conditions. However, we also note that additional studies are needed to determine whether increased concentrations of specific omega-3 oxylipins control the resolution of inflammation in obese humans.

Another way to change oxylipin levels in the circulation in obesity can be via dietary antioxidant intake [93]. It has been reported that systemic COX- and LOX-derived oxylipins are significantly modulated after consuming polyphenol-rich foods. Lara-Guzmán et al. (2020) determined that after eight weeks of consuming filtered coffee with a high polyphenol content, the concentrations of urine 2,3-dinor-15-F2t-IsoP were lower in healthy people [136]. Similarly, red wine consumption in healthy women decreased levels of urinary 2,3-dinor-15-F2t-IsoP and 2,3-dinor-11-β-PGF2α, a pro-inflammatory PG metabolite [137]. A human study showed that polyphenol intake is negatively associated with metabolites of PGD (2,3-dinor-11β-PGF2α) and 15-F2t-IsoPs (2,3-dinor 15-F2t-IsoP and 2,3-dinor-15-epi-15-F2t-IsoP). These findings imply that dietary antioxidants may regulate novel oxylipin metabolites, which may be sensitive indicators of oxidative stress in obesity-related disorders [93].

Diet and its impact on gut microbiota composition may be another potential approach influencing the oxylipin pattern. Changes in gut microbiota composition induced by oxylipins have been recently reviewed. It has been shown that microbiota dysbiosis changes the oxylipin profile in healthy and obese rats, and there is a relationship between different bacterial taxa and oxylipins. For example, there is a strong positive relationship between Proteobacteria and LTB4 [45]. Additionally, it has been claimed that gut bacteria are responsible for postprandial decreases in sEH, an enzyme responsible for metabolizing EETs to less active diols [138]. It has been discovered that the gut microbiota-regulated mucosal regulatory T cells are inhibited by PGE2, a well-known inflammatory mediator [139]. This evidence suggests that it may be possible to prevent obesity by modulating oxylipin production through dietary modulations affecting the gut microbiota [69]. However, further and larger studies are required to investigate the effect of the gut microbiota on oxylipins and obesity.

### 3.2. Insulin Resistance and Diabetes

Diabetes mellitus (DM) is increasing in prevalence and is having an adverse effect on the lives of individuals, medical systems, and entire nations [140]. DM is an endocrine and metabolic disease defined by hyperglycemia brought on by insufficient insulin synthesis or dysfunctional insulin. The condition can give rise to microvascular complications (such as retinopathy, nephropathy, and neuropathy) and macrovascular complications (including hypertension, peripheral vascular disease, cerebrovascular disease, and ischemic heart disease), resulting in a substantial decline in the affected individual’s quality of life. The prevalence of DM has reached a concerning level, resulting in a significant public health issue globally [141,142]. As per the International Diabetes Federation, approximately 537 million individuals aged 20–79 were living with DM in 2021. This figure is anticipated to escalate to 643 million by 2030 and 783 million by 2045, predominantly in nations with low and moderate incomes [143].

DM is characterized by four metabolic abnormalities: impaired insulin production, elevated endogenous glucose, aberrant insulin action, and obesity [144,145,146]. Hyperinsulinemia arises early in the disease, and as β cell failure improves, it impairs glucose homeostasis, resulting in DM. Many people with DM are obese, particularly with excess abdominal fat. Excessive fat tissue accumulation stimulates insulin resistance by activating several inflammatory mechanisms, including an elevated release of free fatty acids and a disruption of adipokine regulation [147,148]. DM also causes oxidative stress by lowering antioxidant capacity and generating increased free radicals. Increased oxidative stress-induced inflammation is another factor involved in the development and pathophysiology of DM [149]. Research results indicate that adipokine dysregulation [150], gut microbiota abnormalities [151], immunological dysregulation [152], and inflammation [153] are major pathophysiological contributors in DM.

Metabolomic research on diabetes has shown alterations in many metabolites, such as lipids, carbohydrates, and amino acids, which indicate disruptions in energy metabolism in T2DM [154,155]. DM induces aberrations in energy metabolism and mitochondrial function by elevating circulating concentrations of branched-chain amino acids and medium- and long-chain acylcarnitines. Nevertheless, a limited amount of research investigates the impact of diabetes on the levels of signaling lipids, such as oxylipins, in the bloodstream [156,157].

It has been indicated that oxylipins have significant implications for obesity and T2DM. Chronic inflammation is linked to obesity, which plays a role in developing insulin resistance and T2DM. 12-HETE, derived from 12-LOX, is a lipid that has been intensively researched. It impairs insulin signaling in adipocytes and impairs β-cell activity by causing inflammation [158]. The augmented synthesis of PGE2 by β-cells may have a vital role in the response of these cells to obesity and insulin resistance in T2DM, particularly when PGE2 and its receptor EP3 are expressed at elevated levels [159]. In obese and inactive women with insulin resistance, it was shown that weight loss reduced the levels of 9,10-diHODE, 12,13-diHODE, and 9,10-diHOME. This finding supported the hypothesis that oxylipin levels, insulin resistance, and T2DM are related [113]. The findings from the Diabetes Autoimmunity Study in the Young (DAISY) indicate that the ARA-related oxylipin 5-HETE is correlated with an elevated risk of type 1 diabetes (T1DM). Furthermore, the study has revealed that children at risk of T1DM displaying heightened levels of oxylipins associated with LA and ALA are linked to a reduced risk of contracting the disease [160]. It was found by Tans et al. that people with T2DM had higher levels of PGF2, PGE2, 15-keto-PGE2, and 13,14-dihydro-15-keto-PGE2 in their plasma compared to control subjects who were either thin or obese [20].

4-Hydroxy-2-nonenal (4-HNE) is generated during the peroxidation of omega-6-PUFA, while 4-hydroxy-2-hexenal (4-HHE) is released as a byproduct of omega-3-PUFA oxidation [161]. The lipid aldehydes are related to impaired insulin action. Diabetic rats have an increase of 4-HNE-adducts in their liver and pancreatic β cells, which has been seen to hinder glucose-induced insulin release in isolated β cells and diminish insulin activity in 3T3-L1 adipocytes and L6 muscle cells [162,163]. Soulage et al. showed that 4-HHE is generated in individuals with T2DM and Zucker diabetic fatty rats, reducing insulin effectiveness in skeletal muscle. 4-HHE levels are increased in the plasma of individuals with T2DM, leading to insulin resistance in vivo and impaired glucose absorption and signaling in skeletal muscle cells in vitro [164].

**Figure 4:**
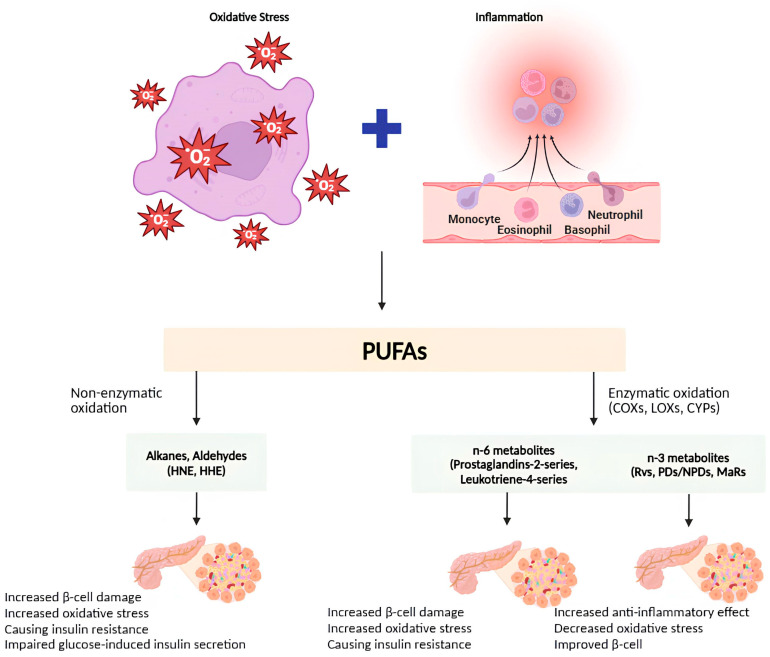
The oxylipins and its impact on diabetes. COX: Cyclooxygenase; CYP: Cytochromes P450; HHE: 4-Hydroxy-2-hexenal; HNE: 4-Hydroxy-2-nonenal; LOX: Lipoxygenase; MaR: Maresin; *n*-3: omega 3 fatty acids; *n*-6: omega 6 fatty acids; PD/NPD: Protectin/neuroprotectin; PUFA: Polyunsaturated fatty acid; Rv: Resolvin; PUFAs: Polyunsaturated fatty acids.

Adipose tissue that is inflamed releases a range of pro-inflammatory compounds, such as chemotactic mediators and cytokines. Additionally, it stimulates the release of free fatty acids [165,166]. COX-2 is upregulated during inflammatory reactions and catalyzes the conversion of AA into PGs and TXs [167]. COX-2 expression leads to inflammation in adipose tissue. Inflammation of adipose tissue also stimulates the activation of COX-2, resulting in insulin resistance and the onset of T2DM [168]. Increasing PGE2 levels, which belong to the COX oxilipin family, also encourage the migration of macrophages into adipose tissue and the hypertrophy of adipocytes. Overall, the elevation in plasma levels of PGF 2α, PGE 2, 15-keto-PGE 2, and 13,14-dihydro-15-keto-PGE 2 among T2DM patients indicates that these oxylipins serve as markers of T2DM resulting from inflammatory adipose tissue in obese people [169,170]. Figure 4 shows the impact of oxylipins on DM. Examining oxylipins as biomarkers and transforming them into approved tests will improve our ability to predict the risk of DM and its underlying processes.

### 3.3. Cardiovascular Diseases

Cardiovascular diseases, such as ischemic heart disease (IHD) and stroke, are the leading global cause of mortality and a major contributor to disability. Cardiovascular diseases continue to affect more than 500 million people globally, leading to 20.5 million deaths in 2021. This accounts for nearly one-third of all global deaths and represents a significant rise from the previously estimated 121 million deaths caused by cardiovascular diseases [171,172]. Cardiovascular diseases can be caused by various risk factors, including lack of physical exercise, excessive intake of sodium, high consumption of alcohol and tobacco, severe obesity, diabetes, abnormal blood lipid levels, high blood pressure, as well as exposure to air pollution in the individual’s living environment [173]. Oxylipins (eicosanoids and docosanoids) are vital for autocrine and paracrine signaling and have essential functions in the cardiovascular system, including angiogenesis, blood vessel permeability, inflammation, and blood coagulation [174].

Nevertheless, oxylipins possess prospective benefits in the management of cardiovascular disorders, as the administration of EPA and DHA through injection results in a decrease in blood triacylglycerol levels [175].

HODEs generated from LA by the action of atypical catalysts present in tissues under pro-oxidant circumstances, such as insufficient blood supply, inflammation, or injury, act as dependable indicators of lipid peroxidation and oxidative damage [176]. Fatty acid-binding protein 4 (FABP4, also known as aP2) plays a crucial part in the process of adipocyte development. The production of FABP4 is controlled by PPAR-γ. FABP4 additionally regulates lipid accumulation in macrophages, and its expression is likewise regulated via PPAR-γ [177]. FABP4 is released from adipocytes by a non-traditional mechanism that is linked to the breakdown of fats. It functions as an adipokine, contributing to the development of insulin resistance and atherosclerosis and thereby metabolism-related low-grade chronic inflammation. Thus, the levels of FABP4 in the bloodstream are positively associated to many symptoms of metabolic syndrome and cardiovascular disease [178].

Furthermore, upregulation of 15-LOX-1 is linked to the characteristic feature of early atherosclerosis and elevated synthesis of 13-HODE, which in turn enhances the expression of CD36 and FABP4 via PPAR-γ activation (13-HODE PPARγ-agonist) and triggers apoptosis. These mechanisms serve as a defense mechanism in the early stages of lesions and can also eliminate lipids and debris from the blood vessel wall, as well as remove cells that are injured or contain a high amount of lipids [179]. 9-HODE has unique effects on macrophages that are different from the effects of 13-HODE. These effects are mediated by a mechanism that does not include PPAR-γ. Within keratinocytes, 9-HODE exhibits pro-inflammatory properties and induces macrophage IL-1β that is facilitated by the G protein-coupled receptor GPR132, also referred to as G2A. Unlike GPR132, 13-HODE does not act as a ligand and primarily has protective properties against inflammation and atherosclerosis. Studies conducted on atherosclerosis-prone animal models have shown that GPR132 has a role in controlling atherosclerosis [180].

Cytochrome P450 1B1 (CYP1B1) and its related cardiotoxic HETEs metabolites have been identified as directly contributing to the development of cardiac hypertrophy [181]. Mid-chain hydroxyeicosatetraenoic acids interact with GPCR, leading to GTPγS coupling and activation of protein kinase C (PKC). PKC, when activated, phosphorylates the MAPK signaling cascade, including extracellular-regulated kinases (ERK), c-Jun NH2-terminal kinases (JNKs), and p38. Phosphorylation of MAPKs activates NF-κB, which subsequently binds to certain DNA sequences called κB. This binding triggers the transcription of target genes that are associated with cardiac hypertrophy among others [182].

20-HETE is a potent vasoconstrictor that plays a role in the progression of hypertension and also causes relaxation. It stimulates the renin–angiotensin system by causing the production of vascular angiotensin-converting enzyme (ACE). 20-HETE stimulates the binding of NF-kB to the promoters of ACE, inducing the transcription of ACE. Thus, the molecular basis for the interaction between 20-HETE and the renin–angiotensin system in the onset and advancement of vascular inflammation, hypertension, and cardiovascular disease is provided [183]. Furthermore, it should be noted that 20-HETE exhibits strong vasoconstrictive properties [184]. One of the functions of the sEH is to break down epoxy fatty acid metabolites (EpOMEs) produced by CYP450 into diHOMEs, which are a class of compounds related to LA. Although diHOMEs have cardioprotective properties at low concentrations, further increases in concentration have been linked to increased vascular permeability. People who die from sepsis are more likely to have high EpOME levels [185]. Increased levels of pro-inflammatory cytokines such as interleukin-6 (IL-6) and TNF-α were significantly associated with diHOMEs levels [185]. Increased oxidative stress was observed in vascular endothelial cells exposed to diHOMEs and EpOMEs [186].

Plasma oxylipins are increasingly used as biomarkers to predict the risk of cardiovascular diseases. Patients with preexisting peripheral arterial disease were the focus of an inquiry into the frequency of coronary and cerebrovascular events [187]. In order to determine the probability of incident acute myocardial infarction (MI), researchers analyzed the serum non-esterified oxylipin profiles of 744 cases and 744 matched controls in a prospective nested case-control study within the Singapore Chinese Health cohort. Hierarchical clustering was used to identify two groups of oxylipins and fatty acids that were determined to be linked with risk. In contrast to AA’s direct relationship with MI risk, TXB2 (not an active metabolite, just a marker of increased AA oxygenation) and 12-HETE (a product of the 12-LOX pathway) showed the opposite effect [188]. Conversely, a smaller retrospective case/control research involving 117 patients with ACS examined the differences between ACS-free individuals and ACS patients who experienced secondary major acute coronary episodes (MACE), with and without follow-up. The concentrations of each of the non-esterified mid chain alcohols of AA (5-, 8-, 9-, 11-, 12-, and 15-HETE), which are byproducts formed either through LOX pathways or autooxidation, were shown to be elevated in both groups of patients with ACS [189]. The CYPepox/sEH pathway has been implicated in the development and progression of cardiovascular disease in a number of studies. Levels of non-esterified EpETrEs, or EETs, were greater in subjects with stable coronary artery disease (CAD) than in healthy people. Obese individuals with CAD also exhibit increased levels of EpETrEs, although to a lesser extent compared to the lean group [190]. Among the OPEs, most of the CYPs that demonstrate epoxygenase activity also have hydroxylase activity, specifically ω/ω-1 hydroxylase [20]. In patients with CAD, the existence of non-esterified 20-HETE, which is generated by the activity of CYPω/ω-1 on arachidonic acid AA, is directly linked to decreased blood vessel function, as evaluated by brachial artery flow mediated dilation. This indicates a potential disturbance in the CYPω/ω-1 pathways. Furthermore, it was demonstrated that the proportions of non-esterified EpETrEs:DiHETrE exhibited an inverse relationship with the chemoattractant MCP-1, suggesting a connection between inflammatory stress and insufficient CYPepox signalling. When comparing the CYPepox and CYPω/ω-1 activity of this enzyme class in various cross-sectional studies, it is evident that the epoxygenase metabolites have a detrimental association with disease progression mediators, while the ω/ω-1 hydroxylase metabolites have a beneficial association with disease progression mediators [46].

**Figure 5:**
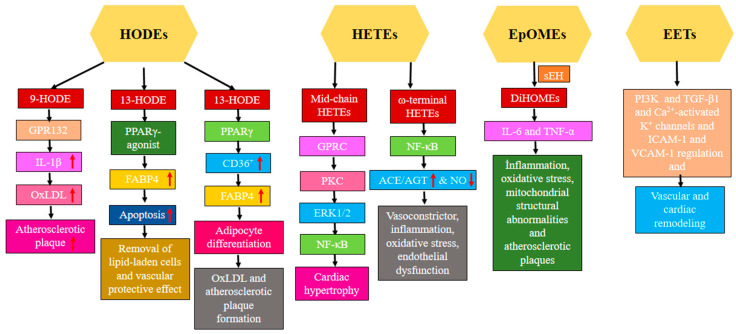
Functions of oxylipins in vascular control. 20-HETE, 20-hydroxy-5,8,11,14-eicosatetraenoic acid; GPR132, G protein-coupled receptor 132; OxLDL, Oxidized Low-density Lipoprotein;IL-1β, Interleukin-1 beta; FABP4, Fatty Acid-Binding Protein 4; PPARγ, Peroxisome proliferator-activated receptor gamma; ERK1/2, extracellular signal-regulated kinase 1/2; PKC, Protein kinase C; NF-κB, nuclear factor-kappa B; NO, nitric oxide; AGT, Angiotensinogen; ACE, angiotensin-converting enzyme; TNF-α, Tumor necrosis factor alpha; IL-6, Interleukin-6; sEH, soluble epoxide hydrolase enzyme; PI3K, hosphatidylinositol-3 kinase; ICAM-1, intracellular adhesion molecule; VCAM-1, vascular cell adhesion molecule 1 1.

In a multi-center observational analysis, 479 patients treated with cardioverter-defibrillators (ICDs) for systolic heart failure were included. The goals of the study were to determine whether there was a correlation between serum oxylipins and mortality and the frequency of ICD shock for ventricular arrhythmias. Five of the six oxylipins, namely 5,6-DHET, 8,9-DHET, and 9,10-diHOME, 17,18-DiHETE, 19,20-DiHDPA associated with ICD shock exhibited increased sEH activity because they are diols. Four specific sEH oxylipins—5,6-DHET:AA, 8,9-DHET:AA, 9,10-diHOME and 17,18-DiHETE:EPA:LA—were associated with mortality as a function of their precursor PUFAs. When sEH activity was high, it produced oxylipins that were associated with ventricular fibrillation or mortality [191]. Patients with mild subcortical small vessel ischemic disease (SVID), defined by a lower white matter hyperintensities (WMH) burden, were compared to patients with severe SVID, who were shown to have a significant burden of WMH in terms of the activity of oxylipin sEH. Increased sEH activity for LA-derived 9,10-diHOME/9,10-EpOME and 12,13-diHOME/12,13-EpOME ratios were among the symptoms observed in individuals with widespread SVID, along with elevated levels of LA-derived 12,13-diHOME in blood serum. The ratios were used to evaluate the activity of sEH in the serum. In addition, a decline in executive function was associated with a LA-generated ratio of 12,13-diHOME to 12,13-EpOME [192]. The observed decrease in the protection of the heart in transgenic mice that had an overexpression of CYP2J2 in their cardiomyocytes was shown to be associated with an age-related buildup of harmful substances called diHOMEs. However, this effect was restored when the activity of sEH was inhibited [193]. Specifically, 5,6-EET, 8,9-EET, 11,12-EET, and 14,15-EET are the four known regioisomers. The anti-inflammatory, vasodilatory, and cardioprotective effects of EETs are well-documented. As another important mechanism in vascular remodeling and atherosclerosis development, they can control the migration of vascular smooth muscle [194]. Nevertheless, EETs work as lipid mediators that trigger a wide range of biological reactions and affect both vascular and cardiac performance. These include inhibiting cell death, reducing inflammation, widening blood vessels, promoting the growth of new blood vessels, inhibiting high blood pressure, and protecting against heart muscle damage caused by either reduced blood flow or other factors unrelated to reduced blood flow [194]. The activation of KATP channels and phosphatidylinositol-3 kinase (PI3K) signaling contribute to the potential benefits on heart health associated with EETs [195]. By inducing oxidative stress, NF-κB, and the PPAR-γ pathway, which play a vital role in controlling the characteristics of cardiac fibroblasts, EET decreased the enlargement of heart muscle cells and the levels of protein complexes involved in remodeling, including collagen I, TGF-β1, and tissue inhibitor of matrix metallopeptidase-1 (TIMMP1) [196]. EETs cause a condition of increased negativity in cells by enhancing the likelihood of Ca2+-activated K+ channels being in an open state. Nevertheless, EETs induce vasodilation and enhance vascular function in many stressful circumstances [194]. E-selectin, ICAM-1, and VCAM-1 expression in pulmonary artery endothelial cells are downregulated by EETs, which modify endothelial cells [197]. Figure 5 illustrates the functions of oxylipins in vascular control.

The potential advantages of oxylipins in the eventual management of cardiovascular disease are clearly evident in clinical trials undertaken with human participants. In a preliminary investigation, it was shown that the injection of EPA and DHA resulted in a reduction of blood triacylglycerol levels, while also having varying effects on cholesterol levels.

### 3.4. Non-Alcoholic Fatty Liver Disease (NAFLD)

NAFLD is defined as a condition in which the amount of fat in the liver exceeds 5% of the liver’s weight as a result of excessive nutrient intake and a sedentary lifestyle without alcohol use. This condition is accompanied by endoplasmic reticulum stress and mitochondrial dysfunction, leading to early fibrosis and inflammation, resulting in NASH. The advanced stage of NASH results in fibrosis, cirrhosis, and liver cancer [198]. The prevalence of NAFLD, which is increasing worldwide, is 25%. NAFLD is more common in individuals with obesity, diabetes, metabolic syndrome, and lipid profile disorders [199]. This situation has led to the term “Metabolic dysfunction-associated fatty liver disease (MAFLD)”. Proposed in 2020, the diagnostic criteria of MAFLD, unlike NAFLD, include the presence of type 2 diabetes, metabolic risk abnormalities, and obesity. Metabolic risk abnormalities include waist circumference, blood pressure, plasma triglycerides, HDL-cholesterol, prediabetes, HOMA-IR score, and plasma high-sensitivity C-reactive protein (hs-CRP) parameters [200].

**Figure 6:**
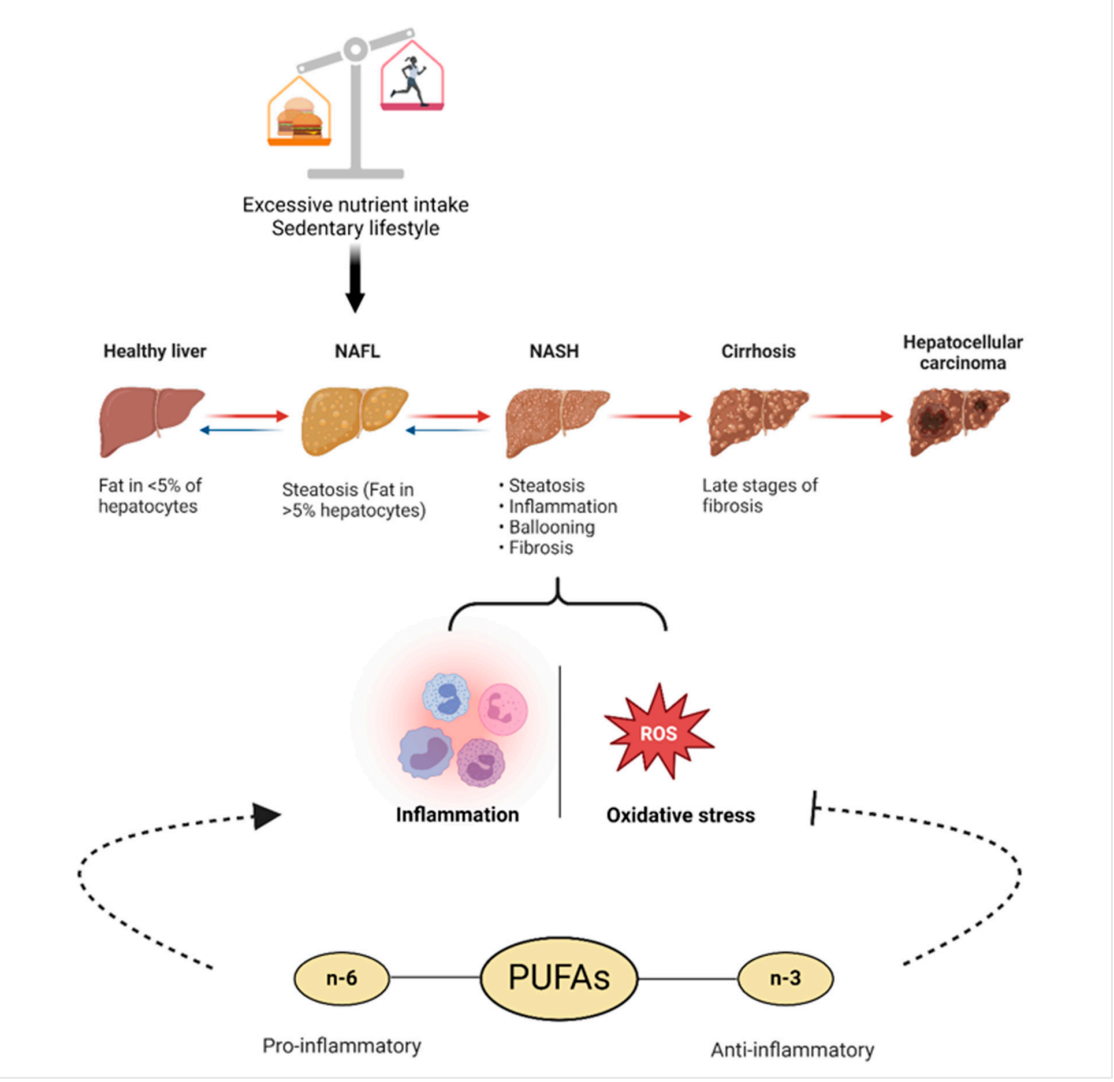
The relationship between NAFLD and oxylipins.

Various biomarkers are used to demonstrate the oxidative stress and inflammatory status present in NAFLD. Superoxide dismutase (SOD), catalase (CAT), myeloperoxidase (MPO), malondialdehyde (MDA), xanthine oxidase (XOD), interleukin-6 (IL-6), hs-CRP, cytokeratin 18 (CK-18), irisin, and plasma oxylipin levels are some of these parameters [53,201,202]. The literature suggests that oxylipins can modulate NAFLD risks and progression and Figure 6 summarizes the relationship between NAFLD and oxylipins. Li et al. [53] analyzed the levels of 52 plasma oxylipin metabolites in adult NAFLD patients and observed that most were increased compared to healthy controls. The elevated levels of tetranor-12-HETE produced by LOX from AA, 20-HETE produced by CYP from AA, 8-HETrE produced by LOX from GLA, and 7-HDoHE produced by LOX from DHA were found to be statistically significant. On the other hand, Mazi et al. [202] revealed that plasma oxylipin levels of NAFLD patients differed according to ethnicity. Compared to Caucasians, AA-derived oxylipins, LOX, and high sEH activities were lower in White Spanish with NASH. Oxylipin levels also varied according to the severity of the disease. PGF2α, LXB4, and MaR1 levels were higher in patients with severe NAFLD than those without NAFLD or mild to moderate NAFLD. Plasma levels of the saturated oxylipins 16-hydroxyl-palmitate (16HPAL) and 3-hydroxyl-myristate (3HMYR) were found to be higher in the early stages of the disease, whereas plasma 12-hydroxyl-estearate (12HEST) levels were found to be higher in the later stages of the disease. As a result, it was suggested that 12HEST and PGF2α plasma levels may be a diagnostic indicator in determining the stage of NAFLD [203].

In a study conducted in young adults, it was observed that as the plasma levels of omega-3 oxylipins 19,20-DiHDPA, 14,15-DiHETE, and 17,18-DiHETE increased, the fatty liver index decreased. Meanwhile, as the plasma levels of omega-6 oxylipins 14,15-EpETrE, 15-HeTrE, 5-HETE, and 8,12-iso-iPF2α-VI increased, the fatty liver index increased [52]. Similarly, a high ratio of (omega-6)/(omega-3) oxylipins is indicative of impaired liver function and an unfavorable cardiometabolic profile in middle-aged adults [49]. In mice, the synergistic effects of high LA intake during the maternal and weaning periods increased the liver omega-6 PUFA ratio. During the weaning period, an LA-rich diet increased the levels of 9-HODE and 13-HODE, which are LOX oxylipins. In addition, a high LA intake showed both pro-inflammatory and anti-inflammatory effects by decreasing anti-inflammatory 8,9-EET levels and increasing plasma concentrations of LXA4, an anti-inflammatory and pro-resolving lipoxin. This study highlights that the interaction between maternal diet and weaning diet, through the omega-6 and omega-3 oxylipins, may play a role in the development of hepatic steatosis in offspring [204].

Lipidome changes in 40 adolescents with Metabolic Dysfunction-Associated Steatotic Liver Disease (MASLD) were examined after a low-free-sugar diet intervention in which free sugar intake was limited to less than 3% for 8 weeks. As a result of the intervention, 8,9-DiHET levels increased; 14,15-DiHET and 8-isoprostane levels decreased. The decrease in 8-isoprostane levels was associated with a decrease in oxidative stress and lipid peroxidation [205]. In a study investigating the relationship between white adipose tissue phenotype and MASLD severity with oxylipin levels in patients who underwent bariatric surgery, it was revealed that the epoxide/diol ratio, which is an indicator of sEH activity, increased with hepatic steatosis [206]. In NAFLD patients, 12 weeks of high-intensity intermittent exercise (HIIT) intervention without dietary intervention did not result in a significant change in plasma oxylipin levels other than 15-F2t-Isprostane compared to the control group. The increase in the level of 15-F2t-Isprostane, an AA-derived oxidative stress indicator, in the exercise group was interpreted as a possible reaction to acute exercise and the result was unclear because this oxylipin level could not be measured in the entire study sample [207].

The level of omega-3 PUFA-derived anti-inflammatory oxylipins was reduced in male Ldlr^−/−^ mice fed a Western diet for 22 weeks to generate the NASH model. When DHA supplementation was given for 8 weeks in addition to the diet of these mice, AA-derived pro-inflammatory oxylipin levels decreased, while C20-22 omega-3 PUFA-derived anti-inflammatory oxylipin levels increased [208]. In the NASH model induced by feeding a Western diet to female Ldlr^−/−^ mice for 38 and 46 weeks, COX1 and COX2 expression and pro-inflammatory omega-6 PUFA-derived oxylipin levels (e.g., PGE2) were increased. On the other hand, the Western diet suppressed DHA-derived oxylipin levels (e.g., 19,20-DiHDPA) and the expression of CYP2C and epoxide hydrolase (Ephx) enzymes involved in fatty epoxide metabolism [209]. Resolvin E1, 9-HETE, and 9-hydroperoxy octadecadienoic acid (9-HpODE) levels were lower in liver tissue of mice supplemented with 1 × 10^9^ cfu Lactococcus lactis subspecies cremoris three times a week for 16 weeks against Western diet-induced metabolic changes [210]. Increased pro-inflammatory oxylipin levels in high-fat diet-induced obese mice were reduced by the addition of 3% krill oil to their diet for 28 weeks. In the liver of krill oil-supplemented mice, EPA concentration increased 10.8-fold and DHA concentration increased two-fold compared to the control group; ARA concentration decreased by 52% [73].

Omega-3-derived SPMs have emerged in the treatment of inflammation and pain in the liver: DHA-derived maresins (MaR1, MaR2), protectins (PD1, PDX) and D-series Rvs (RvD1, RvD2, RvD3, RvD4, RvD5, RvD6); EPA-derived E-series Rvs (RvE1, RvE2, RvE3) and AA-derived lipoxins (LXA4, LXB4) [211,212]. SPMs modulate the body’s response to inflammation by reducing pain sensitivity, macrophage activity, and neutrophil infiltration [211]. Previous studies suggest that low plasma RV levels may play a role in the development of NAFLD [213]; RvD3 attenuates hepatic steatosis through AMPK signaling [214]; RvE1 modulates liver steatosis, fibrosis, and cell proliferation [215]; MaR1 has been shown to increase the polarity of liver macrophages by increasing retinoic acid-related orphan receptor α (RORα) expression and prevent the progression of NASH [216]; while RvD1 and PD1 are protective against NASH by inhibiting Toll-like receptor (TLR4) signaling [217,218].

### 3.5. Chronic Kidney Disease (CKD)

**Figure 7:**
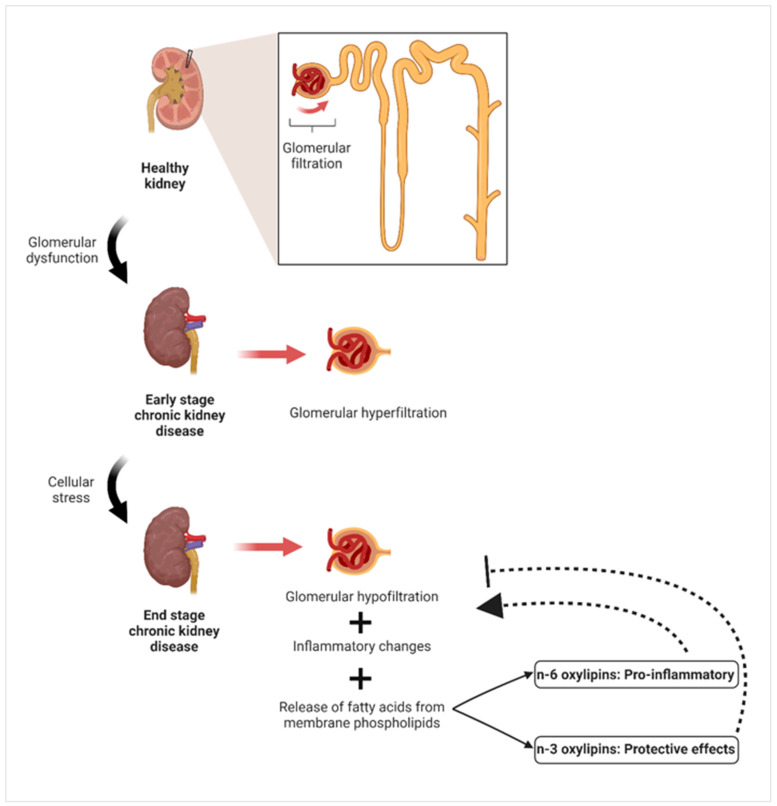
The relationship between CKD and oxylipins.

CKD, with a worldwide prevalence of 9.1%, is defined as abnormalities in the structure and function of the kidney that have been present for at least 3 months and adversely affect health [219,220]. There are three components in the classification of the severity of CKD: an underlying cause, glomerular filtration rate (GFR) category, and albuminuria category [220]. While eGFR is normal in a healthy kidney, in early CKD, eGFR increases with glomerular dysfunction, and filtration increases. In this case, renal function is normal. However, inflammatory changes occur with cellular stress perceived by the podocyte, which acts as a mechanosensor, and fatty acids are released from membrane phospholipids. From these omega-3 and omega-6 derived PUFAs, omega-3 and omega-6 oxylipins are obtained by COX, LOX, and CYP450 enzymes. While omega-3 oxylipins show protective effects, pro-inflammatory signaling of omega-6 oxylipins results in decreased eGFR, glomerular hypertrophy, podocyte detachment, albuminuria, fibrogenesis and ultimately end-stage renal failure (ESKD) (Figure 7) [221].

In a cohort study investigating the role of eicosanoids in the development of ESKD, hydroxyoctadecenoic acid and dihydroxydocosapentaenoic acid (DiHDPE) levels were found to be negatively associated with the development of ESKD, whereas high AA levels were found to be positively associated. With this study, researchers revealed that the eicosanoid profile may have protective effects in the development of kidney disease [222]. Fan et al. [223] showed that EETs regulate epithelial sodium channels in the collecting duct and have renoprotective effects. In addition, deficiency of 20-HETE, which is produced from AA by CYP450 enzymes and involved in myogenic and tubuloglomerular feedback response, causes the development of renal damage. [223,224].

When the oxylipin profile of uremic patients was analyzed, 5,6-DHET, 9(10)-EpOME, 5-HETE and 12(13)-EpOME levels were found to be key markers in differentiating uremic patients and the control group. Plasma 12(13)-EpOME and 9(10)-EpOME levels are negatively correlated with GFR, while plasma 5,6-DHET and 5-HETE levels are positively correlated with GFR [225]. Monirujjaman et al. [226] examined the effect of dietary protein on the oxylipin profile in healthy and kidney diseased mice. No significant change was observed in renal oxylipin levels as a result of intervention with a normal protein diet with 20% of energy from protein, and a high protein diet with 35% of energy from protein for 13 weeks. In the acute kidney injury model of Rund et al. [227], mice were given a standard diet containing 10% fat or a standard diet enriched with omega-3 PUFA containing 1% EPA and 1% DHA for 2 weeks. In the omega-3 enriched group, omega-3 oxylipins (including SPMs) increased. However, this did not affect MCP-1 and IL-6-mediated inflammation and renal damage and preserved tubular function by maintaining the integrity of tubular epithelial cells.

It has been observed in studies that plasma oxylipine levels of hemodialysis patients change [55,228,229,230]. Watkins et al. [55], revealed that plasma EPA levels and EPA-derived 5-HEPE, 12-HEPE, 14,15-DiHETE, and DHA-derived 19(20)-EpDPE oxylipin levels were lower in female hemodialysis patients compared to the control group. To observe the effect of hemodialysis treatment on red blood cells and plasma oxylipin levels, erythrocyte and plasma oxylipin levels were measured before and after hemodialysis [228,230]. Significant changes were observed in the levels of HDHAs, HEPEs, and HETEs, which are LOX oxylipins, in erythrocytes after hemodialysis, whereas most of the CYP pathway metabolites were not affected by hemodialysis [230]. Gollash et al. [229] showed that CYP epoxy metabolites increased after hemodialysis treatment. The levels of 36 oxylipins, including some PGs, isoprostanes, and TXs, were analyzed in the first 6 months following kidney transplantation. The levels of mostly AA-derived PGs and isoprostane 15-keto-15-F2t-IsoP decreased, whereas the levels of PGs 17-trans-PGF3α and 2,3-dinor-11β-PGF2α increased. With this study, it has been demonstrated that oxylipins are useful indicators that can be used in renal transplantation follow-up [231].

sEHs are enzymes involved in the conversion of EETs to DHETs. Since EETs have anti-inflammatory and endothelial dysfunction-preventing effects, sEH inhibition reduces endothelial dysfunction and inflammation with the increase in EETs, and thus renal damage is mitigated. These renal protective effects are mediated by the inhibition of the NFκB signaling pathway and stimulation of the AMPK signaling pathway [232]. Administration of COX-2/sEH inhibitor to Zucker diabetic fatty rats for 8 weeks resulted in 30–80% reduction in renal damage parameters such as albuminuria, nephrinuria, glomerular damage, and renal fibrosis [233]. Similarly, COX-2 inhibition in autosomal dominant polycystic kidney disease improved oxylipin levels in mice and improved renal function and disease progression [234].

## 4. Some Future Directions

Independent of PPAR-γ regulation the modification of FABP4 function by specific antagonists, inhibitors of gene expression (for example microRNA), or neutralizing antibodies could be novel therapeutic approaches for treating diseases such as obesity, DM, cardiovascular disease, and atherosclerosis. Mapping of unidentified FABP4 receptors could also pave the way toward therapeutical approaches and enhance the prevention of diseases or subclinical conditions. Moreover, enhancing the protective EETs pathway could contribute to vasorelaxation and anti-inflammatory and pro-fibrinolytic effects [235].

In summary, oxylipin profile analysis correlates to the existence of certain diseases, for example cardiometabolic disease. By examining time-related changes and trendlines comparing to the same parameters of proper control may reveal further association with the diseases mentioned here or even not mentioned here, for example cancer. Thus, creating a database and analyzing the corresponding quantitative information could be utilized to develop new biomarkers that could be used to monitor disease. Such mathematical models could be applied to evaluate therapeutical approaches including pharmacotherapy, dietetic intervention, or preventive strategies.

## 5. Conclusions

Oxylipins derived from essential fatty acids serve as metabolic regulators, exhibiting powerful effects on several organs and cellular systems. According to the literature, oxylipins have an important role in the pathogenesis of cardiometabolic diseases. Also, they are novel biomarkers for diagnosing cardiometabolic diseases, and in some cases they can be used for monitoring diseases like CKD. Dietary components especially fats and fatty acids can modulate oxylipin production and function.

The impact of dietary omega-3 and omega-6 fatty acids on oxylipin production is crucial in understanding how these dietary fatty acids decrease or increase the risk of cardiometabolic diseases. Oxylipins exert their action through autocrine or paracrine signaling, namely by interacting with peroxisome proliferator-activated receptors to modulate the development and functioning of adipocytes. The main source of oxylipins are high unsaturated PUFAs such as AA, as well as EPA and DHA in high omega-3 dietary situations are more proinflammatory and are associated with atherosclerosis, NAFLD, DM and obesity. On the other hand, an omega-3 fatty acid-enriched diet leads to an increase in the levels of omega-3 EPA-derived and DHA-derived oxylipins, which have anti-inflammatory properties. This rise in anti-inflammatory oxylipins may show favorable effects against cardiometabolic disorders. On the other hand, proinflammatory omega-6 oxylipins may exert the opposite adverse effect.

To fully understand the biological effects of various bioactive lipids, it is essential to conduct a thorough analysis of oxylipins in multiple tissues. This is because different oxylipins, derived from different fatty acids, possess diverse functions and potencies. Such comprehensive profiling allows for a holistic assessment of the overall impact of these lipid derivatives. Advances in analytical methods are contributing to a better understanding of the biological roles of the many oxylipin species that are being discovered. Thus, research comparing the physiological effects of single and combination oxylipins in various organ systems will advance our knowledge of how oxylipins operate in all living things. Specifically, further study into the impact of oxylipins produced from LA and ALA, which constitute the majority of oxylipins in most tissues, would contribute to a greater understanding of their influence on cardiometabolic diseases.

## Figures and Tables

**Table 1:** Role of some oxylipins in obesity.

**Role of AA–Derived Oxylipins**	
PGD2	PGD2 levels increase in mice fed a high-fat diet [92].
PGE2	PGE2 exhibits anti-lipolytic effects, leading to increased adipose tissue mass [91]. COX-2 and PGE2 levels increase in adipose tissue of obese patients [75,76].
PGI2	PGI2 increases adipogenesis via the PGI2 receptor [77]. Body weight gain is reduced in PGI synthase knockout mice feed a high-fat diet [77].
15d-PGJ2	15d-PGJ2 is a metabolite of PGD2 [8]. 15d-PGJ2 shows pro-adipogenic effect [93].
5-HETE	5-LOX expression is upregulated in adipose tissue of obese patients [86]. The activity of 5-LOX is important in chronic and acute inflammation [94]. The inhibition of 5-LOX reduces hepatic macrophage infiltration in obese mice who have a high-fat diet [95]. An increase in 5-HETE promotes oxidative stress and increases reactive oxygen species, which may affect PUFA peroxidation [78].
11-HETE 15-HETE	Increased 11- and 15-HETE concentrations are positively associated with BMI, waist circumference, and serum leptin levels [78].
12-HETE	12-HETE induces inflammation and leukocyte infiltration in obese adipose tissue [89].
LTB4	LTB4 produced via the 5-LOX pathway is related to cytokine release [8]. LTB4 levels are increased in obese mice [79,80]. Inhibition of the LTB4 receptor improves the inflammatory profile by reducing peritoneal macrophage chemotaxis to target organs [79]. Excessive production of LTB4 triggers inflammation by causing increased chemotaxis of circulating B cells to WAT [96]. Leukocytes from obese people exhibit a four-fold increase in LTB4 [84].
20-HETE	20-HETE contributes to rapid weight gain [90]
14,15-EpETrE	EETs block the activation of NF-κB and reduce TNF-α [98]. Stimulation of adipogenesis in vitro and in vivo suppresses adipose-derived EET levels by dysregulating the CYP epoxygenase pathway [98]. Plasma levels of 14,15-EpETrE are positively correlated with adiposity [52]. 14,15-EpETrE levels are lower in fat-1 transgenic mice [57].
**Role of LA-derived oxylipins**	
9-HODE 13-HODE	9-HODE and 13-HODE are associated with oxidative stress, inflammation, and numerous pathological and physiological conditions but the results are contradictory [8,78,92,94,102,103].
12,13-diHOME	12,13-diHOME regulates metabolism and thermogenesis [104]. Plasma 12,13-diHOME concentration was associated with a lower BMI, visceral and total fat mass [19,81].
**Role of EPA-derived oxylipins**	
5-HEPE	5-HEPE plays a role in body energy metabolism and browning of WAT [108]. 5-HEPE promotes BAT activation and browning of WAT by upregulating UCP-1 genes and protein expression [108].
12-HEPE	12-HEPE is synthesized via the 12-LOX pathway [8]. 12-HEPE reduces the risk of obesity by regulating energy homeostasis and fuel utilization [72]. 12-HEPE levels are lower in obese people [72].
**Role of DHA-derived oxylipins**	
14-HDHA 17-HDHA	14-HDHA and 17-HDHA are inflammation-resolving mediators [8]. 17-HDHA is reduced in WAT of obese mice [83]. 14-HDHA and 17-HDHA are lower in the plasma, serum, and leukocytes of obese than in healthy individuals [84].
Rvs, PDs, MaRs	They actively resolve inflammation, protect organs, and stimulate tissue regeneration [8,67,84]. They decrease in response to obesity in various mouse tissues, including WAT, liver, bone marrow, and spleen [83,109]. Increasing the bioavailability of these mediators minimizes inflammation and mediates therapeutic effects [110].

11-HETE: 11-hydroxy-eicosatetraenoic acid, 12,13-DiHOME: 12,13-dihydroxy-9Z-octadecenoic acid, 12-HEPE: 12-hydroxy-eicosapentaenoic acid, 12-HETE: 12-hydroxy-eicosatetraenoic acid, 13-HODE: 13-hydroxy-octadecadienoic acid, 14,15-EpETrE: 14,15-epoxy-eicosatrienoic acid, 14-HDHA: 14-hydroxy-docosahexaenoic acid, 15d-PGJ2: 15-deoxy-Δ12,14-prostaglandin J2, 15-HETE: 15-hydroxy-eicosatetraenoic acid, 17-HDHA: 17-hydroxy-docosahexaenoic acid, 20-HETE: 20-hydroxy-eicosatetraenoic acid, 5-HEPE: 5-hydroxy-eicosapentaenoic acid, 5-HETE: 5-hydroxy-eicosatetraenoic acid, 9-HODE: 9-hydroxy-octadecadienoic acid, AA: Arachidonic acid, BAT: Brown adipose tissue, BMI: Body mass index, COX: Cyclooxygenase, CYP: Cytochrome P450, DHA: Docosapentaenoic acid, EET: Epoxyeicosatrienoic acids, EPA: Eicosapentaenoic acid, LA: Linoleic acid, LOX: Lipoxygenase, LTB4: Leukotriene B4, MaRs: Maresins, NF-κB: Nuclear factor kappa B, PDs: Protectins, PGD2: Prostaglandin D2, PGE2: Prostaglandin E2, PGI2: Prostaglandin I2, Rvs: Resolvins, TNF-α: Tumor necrosis factor alpha, WAT: White adipose tissue.
